# NMR-based metabolite studies with ^15^N amino acids

**DOI:** 10.1038/s41598-019-49208-8

**Published:** 2019-09-05

**Authors:** Benjamin Ramirez, Matthew A. Durst, Arnon Lavie, Michael Caffrey

**Affiliations:** Department of Biochemistry & Molecular Genetics, University of Illinois at Chicago, Chicago, IL 60607 USA

**Keywords:** Biological techniques, NMR spectroscopy

## Abstract

^15^N labeled amino acids are routinely used to label proteins or nucleic acids for study by NMR. However, NMR studies of ^15^N labeled amino acids in metabolite studies have not been pursued extensively, presumably due to line broadening present under standard experimental conditions. In this work, we show that lowering the temperature to −5 °C allows facile characterization of ^15^N-labeled amino acids. Further, we show that this technique can be exploited to measure ^15^NH_3_ produced in an enzyme catalyzed reaction and the transport and metabolism of individual amino acids in mammalian cell culture. With respect to ^13^C-labeled amino acids, ^15^N-labeled amino acids are less costly and enable direct characterization of nitrogen metabolism in complex biological systems by NMR. In summary, the present work significantly expands the metabolite pools and their reactions for study by NMR.

## Introduction

The use of ^15^N-labeled amino acids for the study of biomolecular structures, interactions and dynamics is very well established^1^. In these studies, ^15^N-labeled proteins, RNA or DNA are routinely labeled by preparation in *Escherichia coli* grown in media containing ^15^NH_4_^+^ ^2,3^. In contrast, ^15^N-labeled amino acids have not been used extensively for NMR-based metabolite studies. Presumably this stems from the extensive line-broadening of H^N^ signals due to water exchange in aqueous samples at room temperatures (e.g. the exchange rates of NH_3_ groups with water are >~100/s^4^), which render the H^N^-^15^N group undetectable. On the other hand, ^13^C-labeled amino acids have been used extensively for NMR-based metabolite studies. For example, ^13^C-labeled glutamine is routinely used to characterize TCA cycle intermediates^5^. Recently, Beecher and Larive^6^ demonstrated that the H^N^-^15^N signal of glucosamine is observable at temperatures <0 °C with the addition of organic solvent to obviate freezing. In another study, Millard *et al*.^7^ observed the H^N^-^15^N signals of hydrolyzed amino acids prepared from ^13^C/^15^N-labeled algal and *E. coli* extracts. In the present work, we present NMR-based studies of commercially available ^15^N-labeled amino acids for biochemical analyses and characterization of amino acid transport and nitrogen metabolism in mammalian cells.

**Figure 1:**
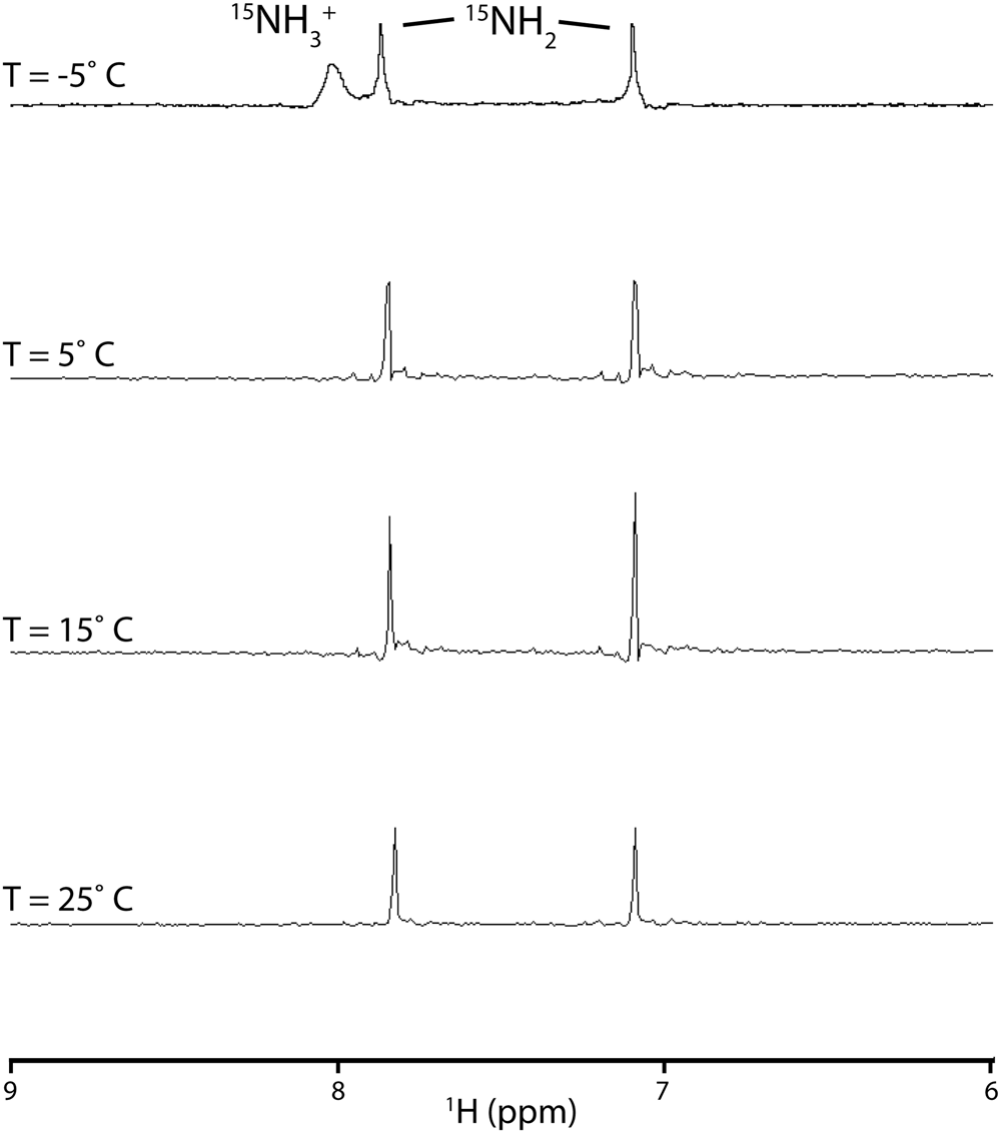
^15^N-filtered 1D spectrum of ^15^N-Asn as a function of temperature. Experimental conditions were 10 mM ^15^N-Asn in 100 mM ^2^H-acetate/pH 4.0, 10% ^2^H_2_O, 20% ^2^H-acetone.

As a first step toward analyzing the potential use of ^15^N-labeled amino acids in NMR-based studies of metabolites, we used ^15^N-labeled Asn, which contains NH_3_^+^ and NH_2_ protons that are in fast and slow exchange, respectively. For these experiments we chose acidic conditions, for which H^N^ exchange rate is minimized, and 20% acetone co-solvent, which enables studies below the freezing point of H_2_O. As shown in Fig. 1, only the NH_2_ groups are readily observed in the ^15^N-filtered NMR spectra at temperatures ranging from 5–25 °C. In contrast, the NH_3_^+^ group is observable at −5 °C, consistent with previous observations of the exchangeable NH_3_^+^ group in glucosamine under similar experimental conditions^6^. Consequently, subsequent studies of exchangeable H^N^ groups were performed under conditions of low temperature and low pH.

**Figure 2:**
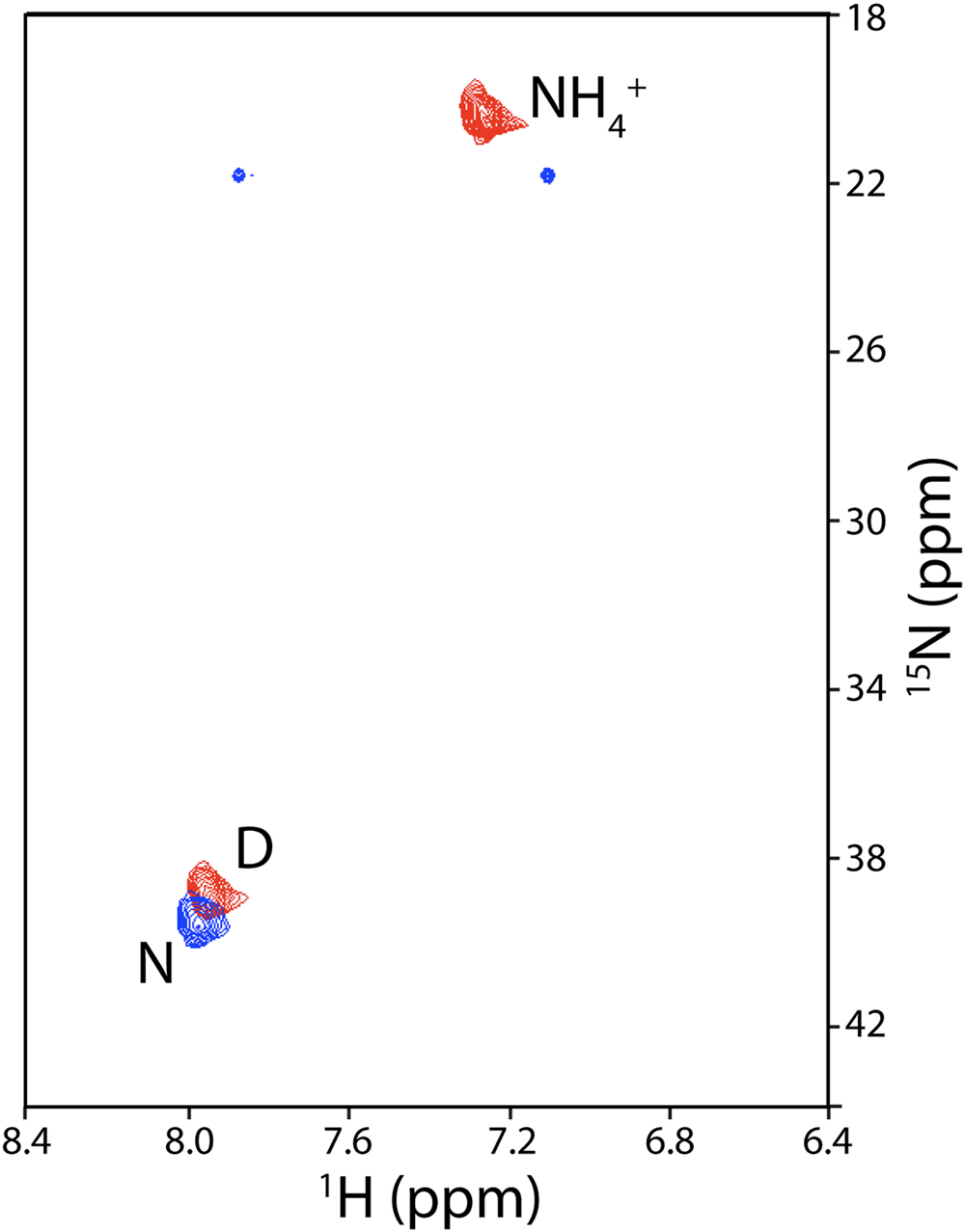
^15^N-edited HSQC of ^15^N-Asn in the presence (red) and absence (blue) of ASNase. Amino acid labels correspond to the one letter code. Buffer conditions were 100 mM ^2^H-acetate/pH 4.0, 10% ^2^H_2_O, 20% ^2^H-acetone.

In the next step, we assayed the ability to detect amino acid metabolism in the media from cultured cells. L-Asparaginase (ASNase) is a FDA-approved therapy for the treatment of acute lymphoblastic leukemia^8^. Moreover, preclinical and clinical data have shown that ASNase is a promising therapy for other hematological cancers and select solid tumors^9–11^. ASNase treatment causes depletion of Asn in the blood by conversion to Asp plus NH_3_. Accordingly, we assayed the metabolism of ^15^N-Asn in cell cultures of LOUCY cells, a model leukemia cell line^12^, as a function of ASNase treatment. As shown by the blue correlations in Fig. 2, the ^15^NH_3_^+^ group of Asn is readily observed in media after 60 min in the absence of ASNase (the NH_2_ groups, which are normally observed at ~115 ppm are relatively weak and appear at an incorrect ^15^N chemical shift in this spectrum due to spectral aliasing). As shown by the red correlations in Fig. 2, treatment with ASNase for 60 min results in quantitative transformation of ^15^N-Asn to ^15^N-Asp and ^15^NH_4_^+^ and thus we are measuring the activity of the enzyme in the media (NH_3_, the product of ASNase catalysis of Asn, is in equilibrium with NH_4_^+^, which is the predominant species at acidic pH).

**Figure 3:**
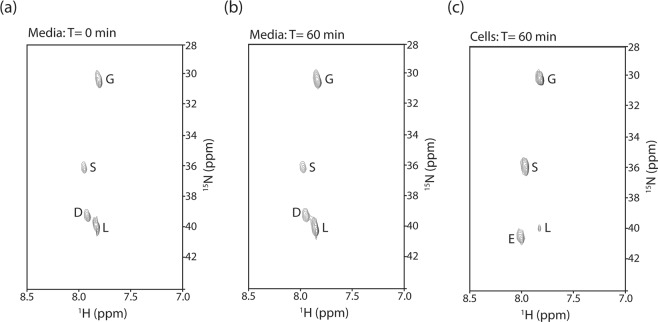
Metabolite profiles of ^15^N-labeled Asp, Gly, Leu and Ser added to LOUCY cells, as assayed by ^15^N-edited HSQC of the media at time = 0 (**a**), the media at time = 60 min (**b**) and the cell extract at time = 60 min (**c**). Amino acid labels correspond to the one letter code. In all experiments, the buffer conditions were 100 mM ^2^H-acetate/pH 4.0, 10% ^2^H_2_O, 20% ^2^H-acetone.

Lastly, we assayed the ability to characterize amino acid transport in cell culture by ^15^N NMR. For these experiments a mixture of 4 ^15^N-labeled amino acids (Asp, Gly, Leu and Ser) was added to the media and the presence of ^15^N-labeled groups was assessed in the media and LOUCY cell extracts. Consequently, the media samples assay the amino acid stability and the cell extract samples assay the amino acid transport and stability. As shown in Fig. 3a, the ^15^NH_3_^+^ of the 4 amino acids are readily observed in the media at time = 0. As shown in Fig. 3b, the 4 amino acids are still observed after 60 min and there are no new correlations observed suggesting that they are not metabolized in the media. Interestingly, as shown in Fig. 3c, the ^15^N-metabolite profile in the cell extracts taken at time = 60 min was very different from that of the media. First note Gly and Ser are readily observed suggesting that they are being transported into the cells. In contrast Asp and Leu are observed at much lower concentrations, suggesting that they are transported at relatively low efficiency and/or being metabolized into other species. Surprisingly, the cell extracts contain a new ^15^N correlation corresponding to Glu, which is clearly absent from the media, suggesting a intracellular transaminase reaction. We note that aspartate transaminase, which catalyzes the interconversion of Asp + a-ketoglutarate ⇔ oxaloacetate + Glu and is present in a wide variety of tissues^13^, is one potential explanation. Alternatively, the branched amino acid transaminase, which catalyzes the interconversion of Leu + 2-oxoglutarate ⇔ 4-methyl-2-oxopentanoate + Glu and is upregulated in numerous cancers^14,15^, is an alternative explanation.

In summary, we have demonstrated the ability to observe ^15^N-labeled amino acids and ^15^NH_4_^+^, a surrogate for NH_3_, in cell culture by NMR. Based of the results presented herein (Figs 2, 3 and the chemical shifts summarized in Table 1), at least 6 amino acids and NH_4_^+^ are sufficiently resolved in the HSQC spectra to be used in future NMR-based metabolite experiments. We note that signal intensity and linewidth may be expected to be further improved through the use of the HISQC sequence, which removes N_y_H_z_ relaxation during nitrogen evolution, and the use of an external D_2_O standard for the NMR lock signal, which removes cross-peak asymmetry^4^. Nonetheless, we have exploited the HSQC technique at low pH and low temperature to characterize nitrogen metabolism and amino acid transport. Importantly, the observation of a novel transaminase activity in leukemia cells may suggest a potential metabolic vulnerability to be exploited. Previously ^13^C-labeled amino acids have been extensively used to study amino acid metabolism^5^; however, ^15^N-labeled amino acids are ~4X less expensive and enable direct studies of nitrogen metabolism. With respect to Mass Spectrometry (MS) studies, NMR offers a number of advantages including easy quantitation, the ability to re-use samples for other analyses, the ability to identify novel metabolites using other NMR experiments (e.g. COSY, ROESY), and the facile interpretation of data (e.g. a typical LC-MS study of cell extracts yields >10,000 signals versus the few signals observed in the ^15^N-edited HSQC spectrum). Nonetheless, MS is clearly much more sensitive and thus more desirable in sample limited situations (e.g. patient biopsy or the study of metabolites at very low concentrations). Finally, we note that the NMR-based technique could be easily extended to *in vivo* studies of amino acids and nitrogen metabolism. For example, non-toxic ^15^N-labeled compounds, which could include amino acids, nucleotides or ammonium, are readily introduced into animals either in the diet or by IV delivery, as previously shown by MS studies^16^.

**Table 1:** Summary of chemical shifts.

Compound	^1^H^N^	^15^N
Asn	7.98	39.81
Asp	7.94	39.09
Glu	8.00	40.65
Gly	7.81	30.33
Leu	7.82	40.02
Ser	7.95	35.96
NH_4_^+^	7.25	20.46

Experimental conditions were 20% d-acetone, 100 mM d-acetate/pH 4.0 and 10% ^2^H_2_O at −5 °C.

## Methods

### Isotope-labeled compounds

^15^N-labeled amino acids were purchased from CIL; ^2^H-acetate and ^2^H-acetone were purchased from Sigma.

### Preparation of media and cells

For the analysis of media and cell extracts, the T-cell acute lymphoblastic leukemia suspension cell line LOUCY^12^ was used. Cells were harvested and re-suspended to 1*10^6^ cells/mL of fresh RPMI 1640 media supplemented with 10% FBS. Cells were allowed to acclimate to new media for 1 hour in a humidified 37° incubator. In the enzyme treated flasks, 1 IU/mL of Era-TM ASNase^12^ was added. After 1 hour in the incubator, either ^15^N-Asn or a mixture of ^15^N- Asp, Gly, Ser and Leu was added to a final concentration of 500 µM of each. For media samples, 800 µL of media was spun for 5 minutes at 1k RCF at 4 °C. 500 µL of supernatant was transferred to a clean 1.5 mL Eppendorf tubes and stored at −20 °C. 1 hour after adding the ^15^N-amino acids mixture, each flask was harvested, chilled on ice and centrifuged for 5 minutes at 1k RCF. The cells were re-suspended in 1 mL of cold DPBS, centrifuged for 5 minutes at 1k RCF. The supernatant was discarded and the cell pellet and conditioned media were stored at −20 °C.

### Preparation of NMR samples

Samples of media +/− ASNase were prepared directly be diluting media into buffer with final concentrations: 25% media, 20% d-acetone, 100 mM d-acetate/pH 4.0 and 10% ^2^H_2_O. Media with individual ^15^N amino acids was prepared by adding 1 ml of 100% ice cold methanol to 0.2 ml media, incubating on ice for 30 min, centrifugation (15 min at 10k), 4 hours of lyophilization, and suspension in buffer (20% d-acetone, 100 mM d-acetate/pH 4.0 and 10% ^2^H_2_O). Cell extracts were prepared by resuspension of cells in 80% ice cold methanol, incubating on ice for 30 min, centrifugation (15 min at 10 k), 4 hours of lyophilization, and suspension in buffer (20% d-acetone, 100 mM d-acetate/pH 4.0 and 10% ^2^H_2_O).

### NMR experiments

NMR experiments were performed on a Bruker AVANCE 800 MHz spectrometer with a liquid nitrogen cooling unit to enable studies below 5 °C and using a TXI room temperature probe. For detection of H^N^-^15^N correlations, a standard HSQC sequence with water suppression by flip back pulses was employed on samples in 3 mm NMR tubes with a final volume of 200 µL. Total experimental times ranged from 0.5 to 2 hours. Chemical shifts were calibrated using DSS and indirect referencing of ^15^N^17^. The effects of low temperature and acetone on the chemical shifts were observed to be minimal (<0.04 ppm). Assignments for the ^1^H^N^-^15^N correlations were obtained from the analysis of individual reference compounds (^15^N-Asn, ^15^N-Asp, ^15^N-Glu, ^15^N-Gly, ^15^N-Ser, ^15^N-Leu and ^15^NH_4_Cl).
